# Electroviscous effect on fluid drag in a microchannel with large zeta potential

**DOI:** 10.3762/bjnano.6.226

**Published:** 2015-11-24

**Authors:** Dalei Jing, Bharat Bhushan

**Affiliations:** 1 School of Mechanical Engineering, University of Shanghai for Science and Technology, Shanghai 200093, P. R. China; 2 Nanoprobe Laboratory for Bio- & Nanotechnology and Biomimetics (NLB2), The Ohio State University, 201 W. 19th Avenue, Columbus, OH 43210-1142, USA

**Keywords:** electroviscous effect, microchannels, pressure-driven flow, slip length, zeta potential

## Abstract

The electroviscous effect has been widely studied to investigate the effect of surface charge-induced electric double layers (EDL) on the pressure-driven flow in a micro/nano channel. EDL has been reported to reduce the velocity of fluid flow and increase the fluid drag. Nevertheless, the study on the combined effect of EDL with large zeta potential up to several hundred millivolts and surface charge depenedent-slip on the micro/nano flow is still needed. In this paper, the nonlinear Poisson–Boltzmann equation for electrical potential and ion distribution in non-overlapping EDL is first analytically solved. Then, the modified Navier–Stokes equation for the flow considering the effect of surface charge on the electrical conductivity of the electrolyte and slip length is analytically solved. This analysis is used to study the effect of non-overlapping EDL with large zeta potential on the pressure-driven flow in a microchannel with no-slip and charge-dependent slip conditions. The results show that the EDL leads to an increase in the fluid drag, but that slip can reduce the fluid drag. When the zeta potential is large enough, the electroviscous effect disappears for flow in the microchannel under a no-slip condition. However, the retardation of EDL on the flow and the enhancement of slip on the flow counteract each other under a slip condition. The underlying mechanisms of the effect of EDL with large zeta potential on fluid drag are the high net ionic concentration near the channel wall and the fast decay of electrical potential in the EDL when the zeta potential is large enough.

## Introduction

With the development of advanced fabrication techniques, micro/nano electro-mechanical systems (MEMS/NEMS) have been realized and widely used. As a significant branch of MEMS/NEMS, micro/nanofluidic systems incorporating micro/nano pumps, valves, mixers, and channels have wide applications, such as micro heat exchangers, drug delivery systems, and lab-on-a-chip bioanalysis [1–2]. Understanding the fundamental mechanisms of the micro/nano fluid flow has inspired wide scientific interest in order to accomplish the manipulation and transportation of micro/nano fluid flow in these micro/nano fluidic devices. Theoretical and experimental studies show that some interfacial properties, such as surface charge, boundary slip, nanobubble and surface roughness, which can be neglected in macroscale fluidics, are believed to significantly affect the micro/nano fluid flow [3–13].

When a droplet of certain liquid contacts with a solid surface, the solid–liquid interface can become spontaneously charged based on different mechanisms, such as adsorption of ions or deionization [5,14–16]. The charged solid–liquid interface affects the ion distribution and causes local net charge in the liquid. Because of the electrostatic interaction, the counter-ions (ions having the opposite charge of the charged interface) are attracted to the interface and the co-ions (ions having the same charge as the charged interface) are repelled from the interface. As a result, the adsorbed layer, a thin layer consisting of immobile ions strongly attracted to the solid surface because of electrostatic interaction, is formed next to the solid surface. Above that, the diffuse layer, a thin layer consisting of mobile ions, is formed because of loose electrostatic force and thermal diffusion. Both the adsorbed layer and the diffuse layer constitute an electric double layer (EDL) [5,14–16]. To characterize the EDL, zeta potential is defined, and it refers to the electrical potential at the shear plane separating the immobile fluid layer strongly attracted to the solid surface from the rest of the liquid. The magnitude of zeta potential is reported to be up to several hundred millivolts [17–19]. For example, Ren et al. [19] found that the zeta potential of the deionized ultra-filtered (DIUF) water/silicon interface is high, up to −245 mV. In the EDL, the electrical potential exponentially declines, as a function of distance to the solid surface. Debye length κ^−1^ is the characteristic thickness of EDL, and is given by [5],

[1]
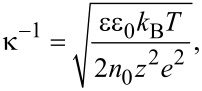


where ε is the dielectric constant of the electrolyte, ε_0_ is the vacuum permittivity, *k*_B_ is the Boltzmann constant, *T* is the absolute temperature, *n*_0_ is the bulk ionic concentration of the symmetric electrolyte, *z* is the valence of the ions, and *e* is the elementary charge.

**Figure 1:**
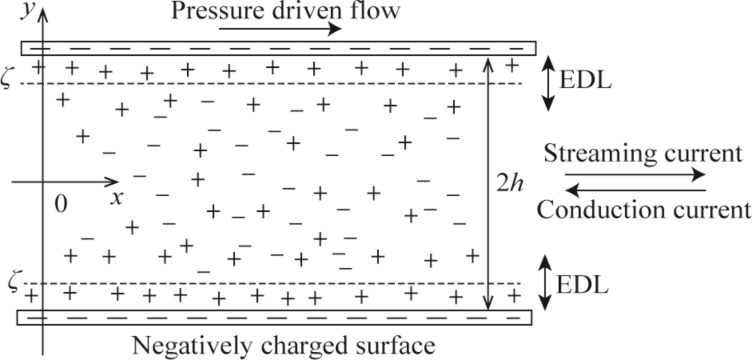
Schematic of the electroviscous effect in a microchannel formed by two infinitely large parallel plates with a distance of 2*h*.

When liquid flows through a channel whose typical dimension is of similar order as the EDL thickness characterized by the Debye length, under the external driven-pressure, the net ion charge in EDL move towards the end of the channel. This results in an electrical current, called streaming current, and a potential difference between the two ends of the channel, called streaming potential. As a result, the streaming potential generates an electrical current, called conduction current, in the direction opposite to the fluid flow. This drives the fluid moving in the opposite direction of the external pressure. The final effect of EDL formed by the charged channel wall applies an electrical force on the fluid flow, reduces the velocity of the fluid flow, and increases the fluid drag. This phenomenon is known as the electroviscous effect [5]. A schematic of the electroviscous effect in a microchannel formed by two infinitely large parallel plates with negative charge is shown in Figure 1.

The electroviscous effect on the fluid flow over the micro/nanoscale has been widely studied. Li [5] reported results of systematical study on the electroviscous effect in microchannels and nanochannels. Yang and Kwok [20] studied the effect of EDL with small zeta potential and slip on fluid flow in a microcircular channel. Soong and Wang [21] studied the effect of non-overlapping EDL with asymmetric small zeta potential on the mass and heat transfer of the fluid flow in the microchannel using the Debye–Hückel approximation. Soong et al. [22] studied the electroviscous effect of EDL on fluid flow in a hydrophobic microchannel with slip-dependent zeta potential. Ban et al. [23] studied the effect of the overlapped EDL with symmetric zeta potential on the electrical conductivity and pressure drop of the pressure-driven flow in a one-dimensional microchannel with no-slip condition. Jing and Bhushan [13] studied effect of the non-overlapping EDL and the overlapped EDL on pressure-driven flow in a microchannel with the charged-dependent slip condition.

At the beginning phase of the study on the electroviscous effect, the linear Poisson–Boltzmann equation was widely used to simplify the analysis, however, the linear Poisson–Boltzmann distribution obtained by the usage of the Debye–Hückel approximation can only be met under the assumption of small zeta potential (usually, the magnitude of zeta potential is smaller than 25 mV) [5,20,24–27]. When the zeta potential of the EDL is large enough, the Debye–Hückel approximation is not valid, and the nonlinear Poisson–Boltzmann should be used to describe the potential and ion distribution in the EDL. Derivation of the analytical solution of the nonlinear Poisson–Boltzmann equation for the potential and ion distribution makes it possible to study the effect of EDL with large zeta potential on the pressure-driven flow in a micro/nano channel [28–30]. For the electroviscous effect in a microchannel with high zeta potential up to several hundred millivolts, Elazhary and Soliman [29] studied the effect of EDL with high zeta potential on the fluid flow and heat transfer in a microchannel with no slip condition. Mondal et al. [30] studied the effect of overlapping EDL with high zeta potential on the velocity field of combined electroosmotic and pressure-driven flow in a microchannel with no slip condition.

In addition, the electrical conductivity of the electrolyte is related to the ionic concentration of the electrolyte, thus, the ions redistribution caused by the charged solid–liquid interface results in the change of the electrical conductivity, and changes the electrical body force applied on the pressure-driven flow by affecting the conduction current. The change in the electrical conductivity is more significant for the EDL with large zeta potential [23,31]. So, the variation in the electrical conductivity should be considered when studying the electroviscous effect.

Further, there is a complicated coupling relationship between the surface charge and boundary slip at the solid–liquid interface. Joly et al. [32] theoretically analyzed the effect of surface charge on the slip and established a mathematical model using molecular dynamics (MD) simulations. They found that a large surface charge density results in a smaller slip. Jing and Bhushan [13] experimentally studied the coupling relationship between the surface charge and the slip of a smooth and hydrophobic octadecyltrichlorosilane (OTS) sample immersed in deionized water and saline solutions using the colloidal probe atomic force microscopy technique. They also found that an increasing surface charge density results in a decreasing slip length. Thus, the coupling between the surface charge and slip should be considered when study the combined effect of EDL and slip on the micro/nano flow.

Although there have been studies on the electroviscous effect in a microchannel with high zeta potential, the study on the combined effect of EDL with high zeta potential up to several hundred millivolts and surface charge-dependent slip on the pressure-driven flow is still needed when considering the change in the electrical conductivity, and the underlying physical mechanisms should be investigated. In order to solve these problems, in this paper, the analytical solutions of the nonlinear Poisson–Boltzmann equation for the electrical potential and ion distribution in the non-overlapping EDL are first obtained without the usage of the Debye–Hückel approximation, and then the effect of EDL on the electrical conductivity and the slip length is analyzed. Then, considering the change in the electrical conductivity and slip length, the effect of EDL with high zeta potential up to several hundred millivolts on the pressure-driven flow is introduced into the Navier–Stokes equation to study the electroviscous effect on the fully developed flow in a one-dimensional parallel-plate microchannel formed by two parallel plates. Both no slip condition and charge-dependent slip condition at the channel walls are investigated. The underlying fundamental mechanisms of the electroviscous effect induced by EDL with high zeta potential are analyzed.

## Theoretical model

A one-dimensional microchannel formed by two parallel smooth plates with a separating distance of 2*h*, as shown in Figure 1, is studied. Here, the height of the microchannel is assumed to be much larger than the Debye length of the liquid, so the EDLs formed separately at the charged upper and bottom channel walls do not overlap with each other. In addition, the upper and bottom walls are assumed to be negatively charged and have the same zeta potential *ζ*. Because the channel wall is negatively charged, so the counter-ions, ions with positive charge (the symbol "+" in the figure) are attracted to the interface, and the co-ions, ions with negative charge (the symbol "−" in the figure) are repelled from the interface. The fluid flow is assumed to be 1:1 symmetric electrolyte in the laminar state, and only the fully developed flow is studied. Figure 1 also shows the coordinate system used in the modeling.

### Electrical potential and ion distribution

Under the above assumptions, the governing equations used to describe the electrical potential and ion distribution in the non-overlapping EDL are given as

[2]
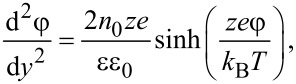


[3]
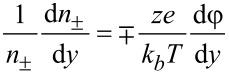


where φ is the electrical potential, *y* is the coordinate axis, *n*_+_ and *n*_−_ represent the ionic concentration of counter-ions and co-ions, respectively.

For the non-overlapping EDL, the boundary conditions of electrical potential and ionic concentration are given by

[4]
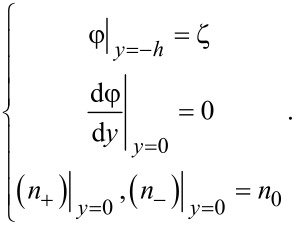


Then, the dimensionless potential and the dimensionless ionic concentration can be obtained as

[5]
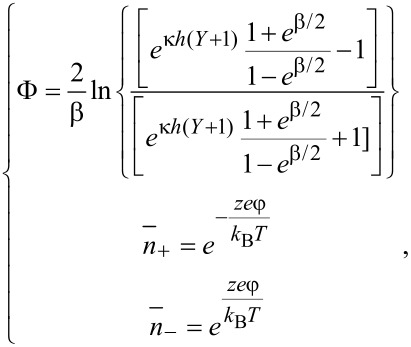


where Φ is the dimensionless electrical potential, and 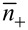 and 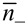 are the dimensionless ionic concentration of counter-ions and co-ions, respectively. In addition, the dimensionless parameters are defined as Φ = φ/ζ, 
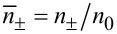
, β = *ze*ζ/*k*_B_*T*, *Y* = *y*/*h*.

Equation 5 gives the analytical solution of the nonlinear Poisson–Boltzmann equation. It can be used to study the potential and ion distribution of the non-overlapping EDL with a large zeta potential.

### Average electrical conductivity

After obtaining the electrical potential and ion distribution in the EDL, it is possible to analyze the variation in electrical conductivity of the electrolyte, which is essential to study the electroviscous effect. The electrical conductivity of a symmetric electrolyte is the function of the ionic concentration and can be given by [23,33],

[6]
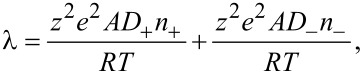


where *A* is the Avogadro constant, *D*_+_ and *D*_−_ are the diffusion constants of counter-ions and co-ions respectively, and *R* is the universal gas constant.

From Equation 5 and Equation 6, the electrical conductivity, which is related to the ionic concentrations of counter-ions and co-ions, is a position-dependent parameter because of ion redistribution in the EDL caused by the surface charge at the solid–liquid interface. To analyze the electrical force applied on the flow, the position-dependent electrical conductivity should be integrated across the microchannel to obtain the effect electrical conductivity, that is, the average electrical conductivity of the electrolyte,

[7]
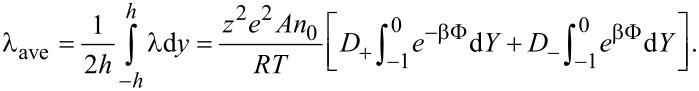


Here, the dimensionless average electrical conductivity, λ_ave_/λ_0_ is defined to analyze the effect of EDL on the electrical conductivity. In this definition, λ_0_ = *z*^2^*e*^2^*An*_0_(*D*_+_ + *D*_−_)/*RT*, and it is the original bulk electrical conductivity of the electrolyte.

### The flow field in the microchannel

The governing equation of the electroviscous effect is given by the following modified Navier–Stokes equation,

[8]
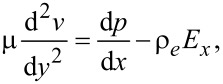


where μ is the dynamic viscosity of the electrolyte, *v* is the velocity of the fluid flow in the microchannel, d*p*/d*x* is the driving pressure gradient, ρ*_e_* = *ze*(*n*_+_ − *n*_−_) is the local net charge density in the EDL, and *E_x_* is the electrical field strength.

The modified Navier–Stokes equation gives the velocity boundary conditions

[9]
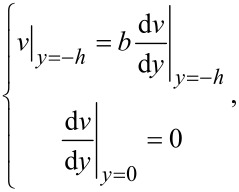


where *b* is the charge-dependent slip length.

The effect of surface charge on the slip length of the solid–liquid interface can be expressed as [32],

[10]
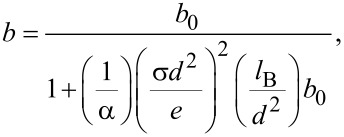


where *b*_0_ is the original slip length without the effect of surface charge, α is a numerical factor, *d* is the equilibrium distance of Lennard-Jones potential, and *l*_B_ is the Bjerrum length. Because the zeta potential can be used to characterize the surface charge density, the Equation 10 can be transferred as

[11]
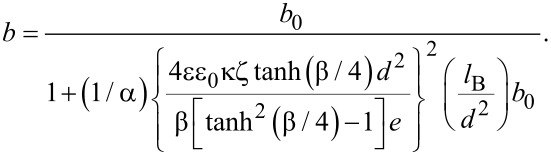


Then, the velocity field and the flow rate of the fluid flow in the microchannel can be expressed as

[12]
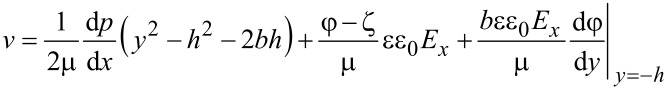


and

[13]
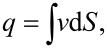


where, *S* is the cross-sectional area of the channel.

Here, the electrical field strength is given by the balance of streaming current and conduction current as [5,13],

[14]
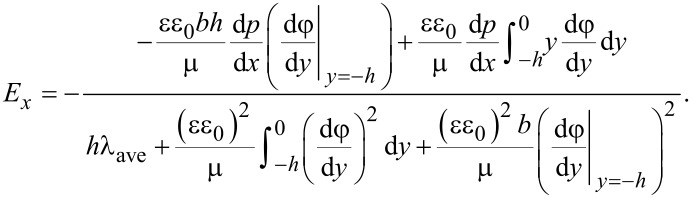


To study the electroviscous effect on the fluid flow and the fluid drag, two parameters, dimensionless velocity *V* and dimensionless flow rate *Q*, are defined as

[15]
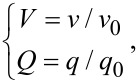


where *v*_0_ is the velocity of the fluid flow at the center of the microchannel without slip and EDL, *q*_0_ is the flow rate of flow in the microchannel without slip and EDL.

## Results

After establishing the model of the effect of the non-overlapping EDL on the pressure-driven flow in the microchannel, some properties of the electrolyte and parameters are chosen to carry out the analysis. Here, deionized (DI) water is chosen as the electrolyte, and the diffusion constants of OH^−^ and H^+^ are assumed to be the same. Table 1 shows the properties and parameters used in the analysis [23,34].

**Table 1:** The properties of DI water and parameters.

property	symbol	value

dynamic viscosity (mPa·s) [34]	μ	0.98
diffusion constant (m^2^/s) [23]	*D*	7.28 × 10^−9^
vacuum permittivity (F/m) [34]	ε_0_	8.85 × 10^−12^
dielectric constant [34]	ε	80
bulk ionic concentration (m^−3^)	*n*_0_	6.02 × 10^20^
pressure gradient (N/m^3^)	d*p/*d*x*	−1 × 10^6^
temperature (K)	*T*	298

### Electrical potential and ion distribution in EDL

**Figure 2:**
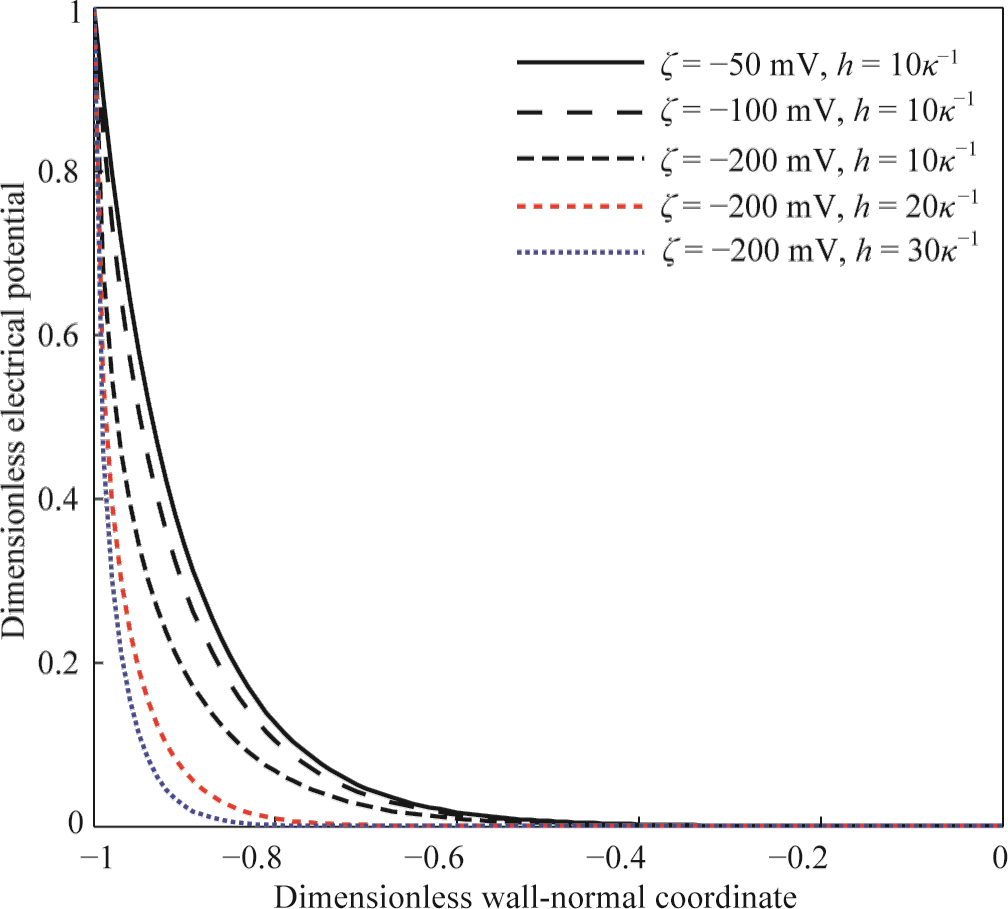
The dimensionless electrical potential as a function of dimensionless wall-normal coordinate in the non-overlapping EDL.

**Figure 3:**
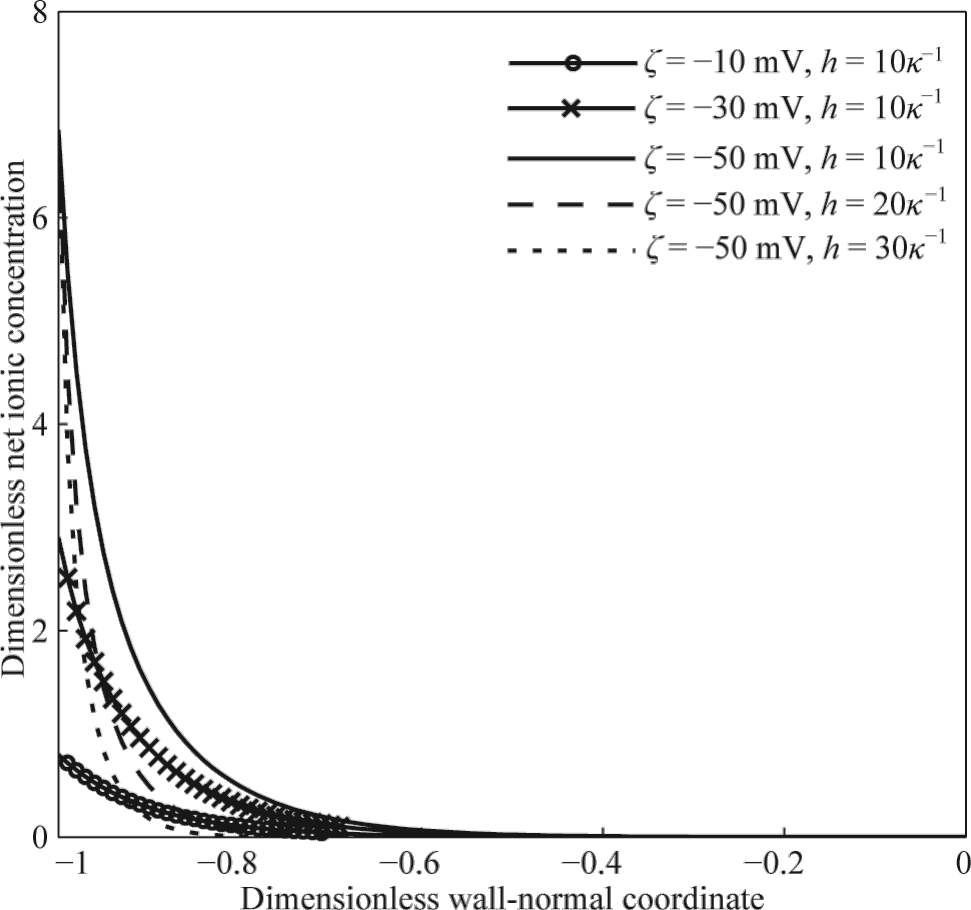
The dimensionless net ionic concentration as a function of dimensionless wall-normal coordinate in the non-overlapping EDL.

Figure 2 and Figure 3 show the distributions of dimensionless electrical potential and dimensionless net local ionic concentration in the non-overlapping EDL. Since the distribution is symmetrical along the *x*-axis, only half of the curves are given in Figure 2 and Figure 3. From the results shown in Figure 2 and Figure 3, under the current parameters, the dimensionless electrical potential and dimensionless net local ionic concentration in the microchannel reduce gradually to zero at the center of the microchannel, which means that the two separate EDLs formed at the lower and upper channel walls do not overlap with each other. This is because the channel height is much larger than the Debye length of DI water. For the microchannel with the same height, both the dimensionless electrical potential and the dimensionless net local ionic concentration show a faster decay with a larger zeta potential at the wall. This means that many more counter-ions are attracted to the channel wall and there is a larger net local ionic concentration near the channel wall, and the two separate EDLs are more difficult to overlap with each other. Similarly, for the channel with a larger height and the same zeta potential at wall, both the dimensionless electrical potential and the dimensionless net ionic concentration show a faster decay with the dimensionless height.

### Average electrical conductivity

**Figure 4:**
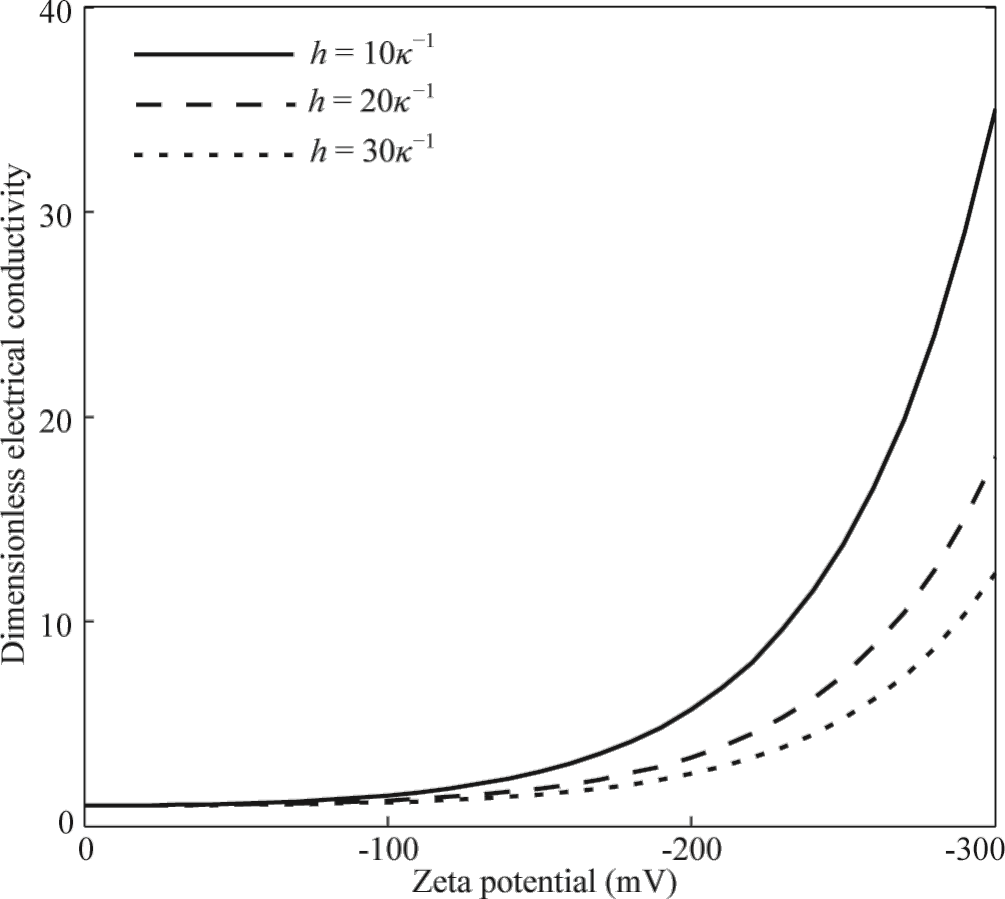
The effect of zeta potential on the average electrical conductivity in the non-overlapping EDL.

Figure 4 shows the effect of the non-overlapping EDL on the dimensionless average electrical conductivity of DI water. According to Equation 6, the electrical conductivity of the electrolyte is related to the ionic concentration, and a larger ionic concentration leads to a larger electrical conductivity. This mechanism is used to explain the variation in the average electrical conductivity with the zeta potential and the channel height. From the results shown in Figure 4, a larger zeta potential results in a larger increase in the average electrical conductivity. The reason is that surface charge at the solid–liquid interface results in the ion redistribution of the electrolyte, and a larger zeta potential leads to a larger net ionic concentration in the EDL, as shown in Figure 3. From the results shown in Figure 4, there is a larger variation in the dimensionless average electrical conductivity for the case of smaller channel height under the same zeta potential. This can also be explained by the larger net ionic concentration in a smaller channel, as shown in Figure 4. Under the same zeta potential, a smaller channel height means a larger increase in the net ionic concentration, as shown in Figure 3, and leads to a larger variation in the average electrical conductivity.

### Effect of EDL on flow field

#### The velocity field

**Figure 5:**
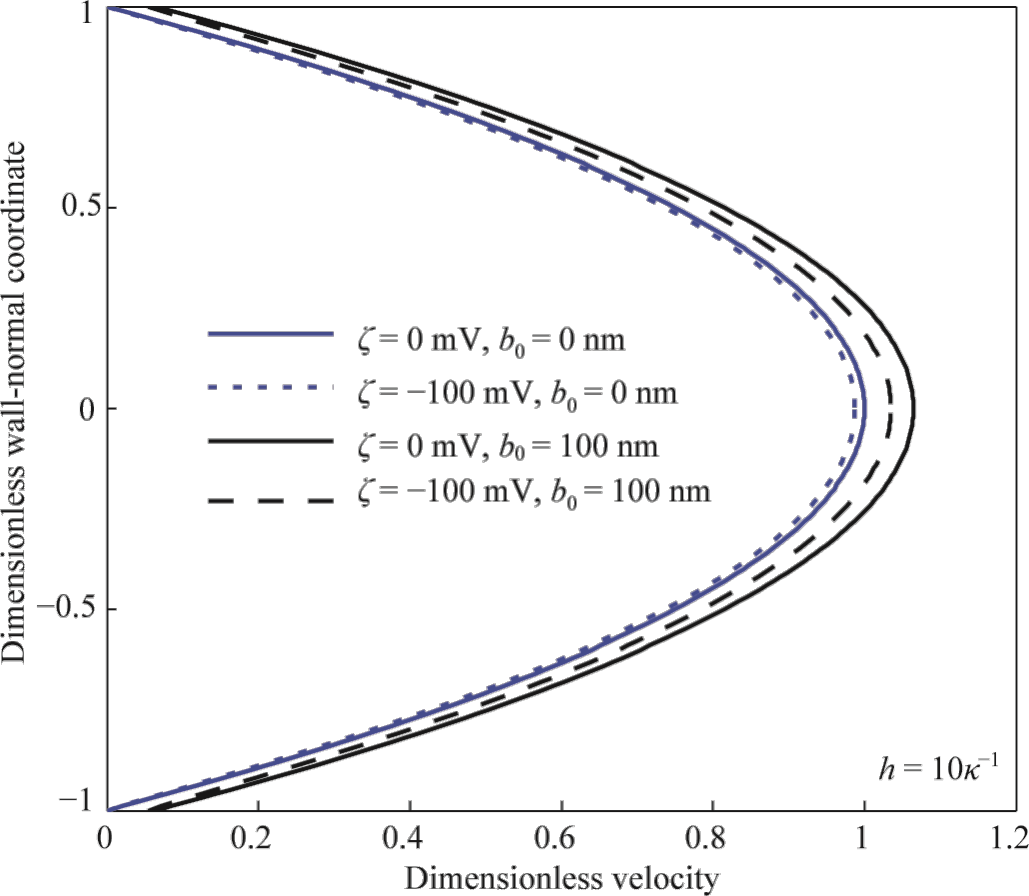
The dimensionless velocity field in the microchannel with different conditions of surface charge and boundary conditions.

After obtaining the electrical potential and ionic concentration in the non-overlapping EDL and the change in the average electrical conductivity and the slip length caused by the surface charge, the velocity field of the pressure-driven flow in the one-dimensional parallel-plates microchannel can be analyzed using Equation 12. Figure 5 shows examples of the dimensionless velocity field in the microchannel with different conditions at the channel walls. Using the velocity field in the case of no slip and no surface charge (the blue solid line) as a reference, the existence of surface charge reduces velocity of the fluid flow (the blue dashes line) because of the electrical force in the opposite direction of the velocity applied on the flow caused by the surface charge-induced EDL, and the existence of slip leads to an increase in velocity (the black solid line). When considering the combined effect of surface charge and slip, there is still an increase in the velocity (the black dashed line) in the case of ζ = −100 mV and *b*_0_ = 100 nm when compared to the reference velocity. It should be noted that there is a counteraction between the effect of surface charge on the flow and the effect of slip on the flow, and it is possible that there is a decrease in the final velocity when considering the combined effect of surface charge and slip. More details will be given in the following section.

#### The fluid drag

**Figure 6:**
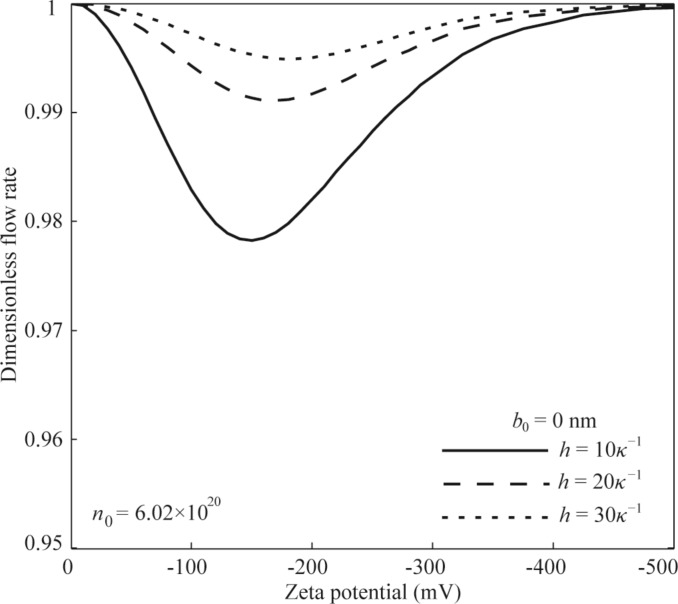
The effect of EDL with high zeta potential on the flow rate of the pressure-driven flow in the microchannel with no-slip condition.

**No-slip condition:** The effect of surface charge-induced non-overlapping EDL on the flow rate of pressure-driven flow in a microchannel with no-slip condition is shown in Figure 6. From the results shown in Figure 6, it can be found that the existence of the EDL results in a decrease in the flow rate, known as the electroviscous effect. It can also be found that the flow rate of the pressure-driven flow first decreases and then gradually increases with the increasing zeta potential. The electroviscous effect disappears when the zeta potential is large enough. Additionally, the flow rate of the pressure-driven flow shows a larger decrease when the channel height is smaller.

**Figure 7:**
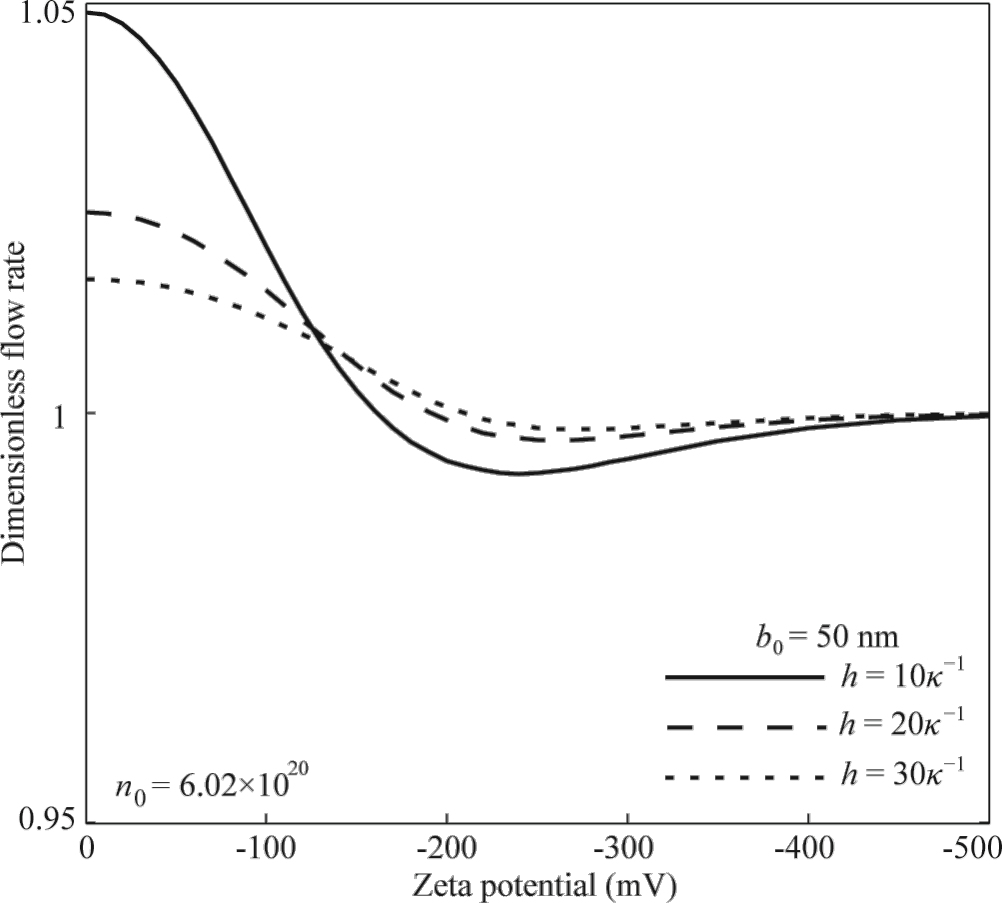
The effect of EDL with high zeta potential on the flow rate of the pressure-driven flow in the microchannel with slip condition.

**Slip condition:** The effect of surface charge-induced non-overlapping EDL on the flow rate of pressure-driven flow in a microchannel with charge-dependent slip condition is shown in Figure 7. From the results shown in Figure 7, the flow rate of pressure-driven flow in a microchannel with slip condition at the channel wall first decreases and then increases with the increasing zeta potential. When the zeta potential is large enough, the retardation effect of surface charge-induced EDL on the flow and the enhancement effect of slip on the flow counteract each other, and the flow rate approaches the flow rate of the pressure-driven flow in a microchannel without surface charge and slip. In addition, when the zeta potential is small enough, the combined effect of slip and EDL leads to an increase in the flow rate when comparing with the flow rate without slip and EDL. However, when the zeta potential is large enough, the combined effect of slip and EDL leads to a decrease in the flow rate. Being similar to the results in the case of no-slip condition, the flow rate shows a larger change when the channel height is smaller.

## Discussion

**Figure 8:**
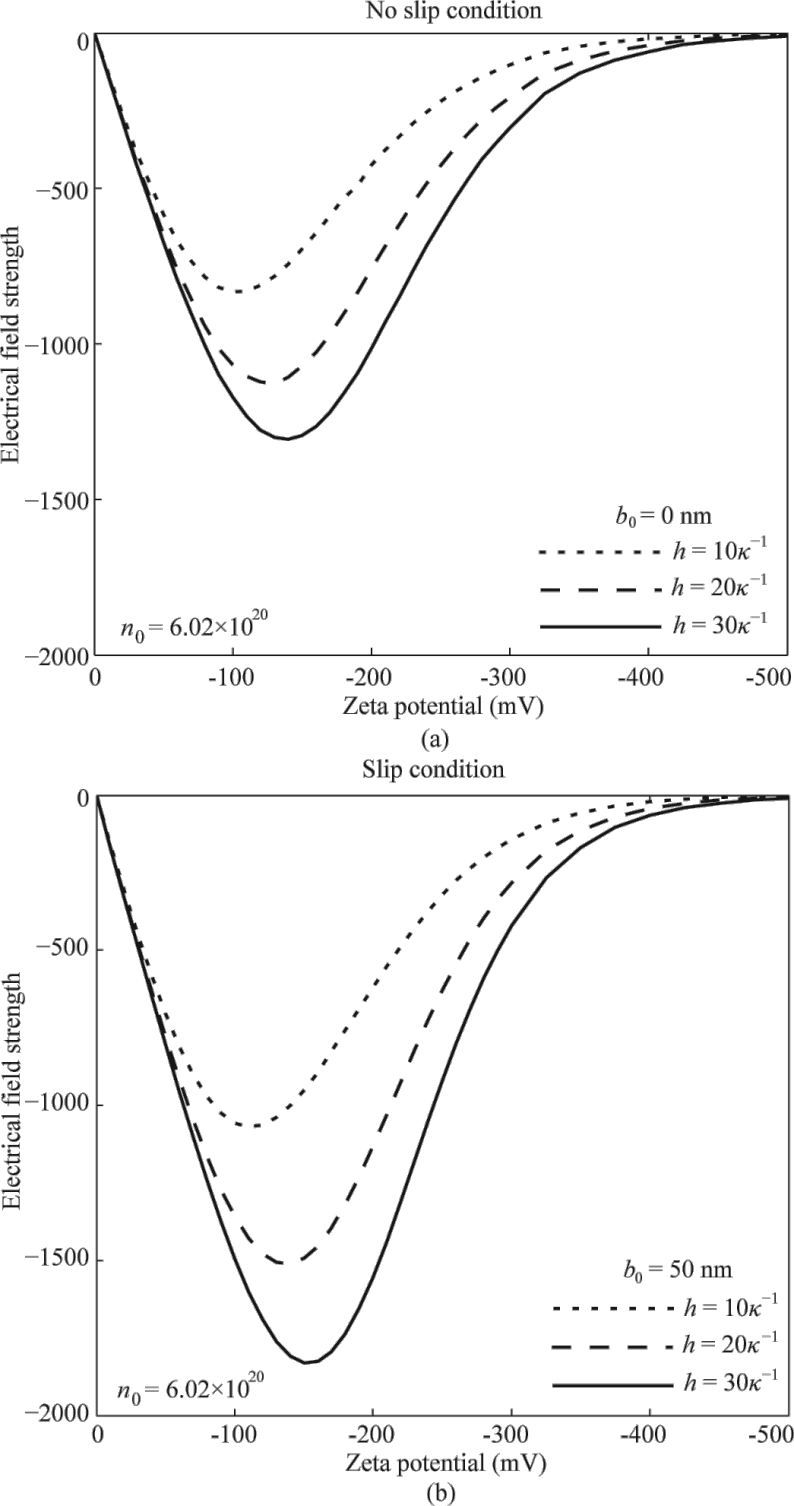
The effect of zeta potential on the electrical field strength of the pressure-driven fluid flow in the microchannel with no-slip condition (a) and slip condition (b).

The effect of surface charge-induced EDL on the flow is achieved by the electrical force applied on the flow (ρ*_e_E_x_*). In order to analyze the electroviscous effect, the change in net local charge density ρ*_e_* and electrical field strength *E_x_* with the zeta potential are needed. From the results shown in Figure 3, ρ*_e_* increases with increasing zeta potential. Figure 8 shows the results of electrical field strength *E_x_*. From the results shown, both for the cases of no-slip and charge-dependent slip conditions, the electrical field strength first increases and then gradually decreases to zero with the increasing zeta potential.

What is the reason for the decreasing electrical field strength with the increasing zeta potential when the zeta potential? According to Equation 14, electrical field strength is related to the potential distribution of the EDL. For the larger zeta potential, there is a larger ionic concentration next to the channel wall and a faster decay for the potential, as shown in Figure 2. This means only a thinner layer next to the channel wall dominates the electrical field strength, so the electrical field strength has a decreasing trend when the zeta potential is increasing. When the zeta potential is large enough, the layer dominates the electrical field strength is narrow enough and the electrical field strength reduces to zero. In additional, when comparing the electrical field strength of no-slip condition and slip condition, the case of slip condition shows a larger electrical strength. The reason is that the existence of slip will increase the velocity of the fluid flow and transportation of ions, which then leads to a larger electrical field strength. The existence of slip will enhance the electroviscous effect.

For the case of no-slip condition, the velocity field of the fluid flow is given by

[16]
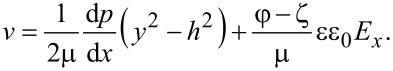


As shown in Figure 8a, when the zeta potential is large enough, electrical field strength gradually reduces to zero. This can be used to explain the disappearing electroviscous effect in the microchannel with a no-slip condition and a large zeta potential. From the point of electrical force applied on the fluid flow, the degree of the electroviscous effect is related to the electrical force applied on the flow, which is determined by the net local charge density ρ*_e_* and electrical field strength *E_x_*. When the zeta potential is small, both the increasing ρ*_e_* and increasing *E_x_* with the increasing zeta potential result in an increasing electrical force applied on the flow and then result in the decreasing flow rate. When the zeta potential is large enough, the effect of decreasing *E_x_* with the increasing zeta potential on the electrical body force overweighs the effect of ρ*_e_*, thus, the electrical force applied on the flow decreases and the flow rate shows an increasing trend with the increasing zeta potential.

For the charge-dependent slip condition, the decreasing slip length with increasing zeta potential based on Equation 11 [13,32] reduces the velocity and the flow rate. For the effect of EDL on the flow rate, it can still be explained by the electrical body force applied on the flow, which is determined by ρ*_e_* and *E_x_*. When the zeta potential is large enough, the effect of decreasing *E_x_* with the increasing zeta potential on the electrical body force overweighs the effect of ρ*_e_*, and this explains the increasing flow rate with the increasing zeta potential, as shown in Figure 8b. In addition, when the zeta potential is large enough, the electrical field strength is close to zero, so the velocity field of the fluid flow is simplified to be

[17]
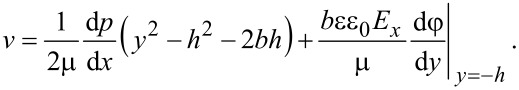


The effect of surface charge induced EDL on the velocity field is dominated by


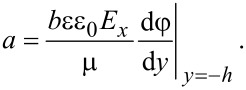


When the zeta potential is large enough, *a* is close to (*bh*/μ)(d*p*/d*x*) because of the larger ionic concentration next to channel wall and the faster decay for the potential, which means


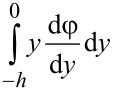


and


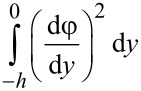


in Equation 14 approach zero. Thus, the retardation of surface charge-induced EDL on the flow and the enhancement of slip on the flow counteract each other.

In all, when the zeta potential is large enough, the electroviscous effect disappears for pressure-driven flow in a microchannel with a no-slip condition, and the retardation effect of EDL on the flow and the enhancement effect of slip on the flow counteract each other for the case of slip condition. The underlying mechanism of these phenomenon is the decreasing electrical field strength and decreasing electrical body force, which is determined by the excessive concentration of ions near the channel wall and the dramatically fast decay of electrical potential in the EDL relating to the large zeta potential.

## Conclusion

In this paper, the nonlinear Poisson–Boltzmann equation is analytically solved to analyze the electrical potential and ion distribution in the non-overlapping EDL with a large zeta potential. Then, considering the effect of non-overlapping EDL on the electrical conductivity and slip length, the effect of non-overlapping EDL with large zeta potential up to several hundred millivolts on the pressure-driven flow is introduced into the Navier–Stokes equation to study the electroviscous effect in a one-dimensional microchannel with no-slip condition and charge-dependent slip condition at the channel walls. The results show that the existence of surface charge leads to the redistribution of ions in an electrolyte and results in an increase in the average electrical conductivity of the electrolyte. Further, the surface charge at the solid–liquid interface can increase the interaction between the liquid and solid, and then reduce the slip length. The surface charge-induced EDL reduces the velocity field of the pressure-driven flow and increases the fluid drag in the microchannel, however, when the zeta potential of the EDL is large enough, the electroviscous effect disappears in the microchannel with no-slip condition. Additionally, the retardation of EDL on the flow and the enhancement of slip on the flow counteract each other for the case of a slip condition. This is because the high net ionic concentration near the channel wall and the dramatically fast decay of electrical potential in the EDL caused by the large zeta potential lead to a narrowing layer dominating the electrical field strength, and then decrease electrical force applied on the fluid flow.

The present study provides a method to control the fluid drag at micro/nano scale by increasing the electrical potential at the solid–liquid interface. This can be accomplished by changing the surface charge density of solid–liquid interface, or applying an external electric field.
